# Normative Values for Londrina ADL Protocol in Healthy Individuals in Age Group of 40–60 Years among Indian Population: A Cross-Sectional Study

**DOI:** 10.1155/2020/8612928

**Published:** 2020-02-18

**Authors:** Uttanoor Sreedevi, Gopala Krishna Alaparthi, Shyam krishnan, Kalyana Chakravarthy Bairapareddy, R. Anand, Vishak Acharya

**Affiliations:** ^1^Department of Physiotherapy, Kasturba Medical College, Manipal Academy of Higher Education, Bejai, Mangalore-575004, India; ^2^Department of Physiotherapy, College of Health Sciences University of Sharjah, Sharjah, UAE; ^3^Department of Pulmonary Medicine, Kasturba Medical College, Manipal Academy of Higher Education, Mangalore-575004, India

## Abstract

**Background:**

Due to increase in the life expectancy and changes related to aging, it is important to assess ADL (activities of daily living) in older adults. However, there is no standardized protocol available to assess ADLs. Considering the limitations of the available tools, a new protocol named Londrina ADL protocol was developed for which normative values are unavailable in different ethnic groups.

**Objective:**

To develop the normative value and reference equation for the Londrina ADL protocol on the basis of anthropometric and demographic variables in healthy individuals in the age group of 40–60 years among the Indian population.

**Methods:**

This cross-sectional study was conducted with 282 healthy individuals of both the genders between the age group of 40 and 60 and they were divided into 2 groups: 40–49 and 50–60. Each subject was made to perform the Londrina ADL protocol twice with a 30 min interval between the two protocols. The protocol is composed of 5 activities and the best out of the 2 performances were recorded.

**Results:**

The mean time taken among 40–49 years by females is 3.50 ± 0.50 min and by males is 3.73 ± 0.43 min. The mean time taken among 50–60 years by females is 4.25 ± 0.20 min and by males is 4.36 ± 0.18 min. The reference equation to predict reference values for the Londrina ADL protocol was as follows: equation (1): Londrina ADL predicted = 1.205 + (0.054 × age (years)) + (0.001 × height (cm)); equation (2): Londrina ADL predicted = 1.374 + (0.054 × age (years)) + (−0.003 × BMI).

**Conclusion:**

The reference equation for the time to complete the Londrina ADL protocol was based on age and BMI as independent variables and can be useful for predicting the performance of healthy individuals.

## 1. Introduction

Functional capacity is defined as “one's maximum potential to perform activities of daily living” such as self-care (bathing, dressing, and eating), leisure, work, and household chores. On the other hand, functional performance is defined as “the physical, psychological, social, occupational, and spiritual activities people actually do in the normal course of their lives to meet basic needs” [1].

In recent years, the consequences of aging have increased in the society due to the worldwide increase in life expectancy. Changes related to aging, obesity, and presence of chronic degenerative diseases, especially cardiopulmonary ones, may be responsible for impairments of mobility, balance, and muscle strength which interfere with the performance of daily life activities. In the age group of 40–60 years, chronic diseases such as chronic bronchitis, emphysema, asthma, bronchiectasis, and occupational lung disease are common. These may lead to loss of independence and poor quality of life. Every individual's tasks and daily routine differ. But walking is an essential component of everyone's daily activities. This made the foundation for the development of functional walk tests that helps to evaluate the mobility, functional capacity, and exercise tolerance of an individual. Therefore, the assessment of ADL (activities of daily living) is of utmost importance in this population [2–5].

In this regard, 6-minute walk test is widely used to assess the functional capacity of an aging individual [6, 7]. Although it reflects limitations in the performance of daily living activities [8], the 6-minute walk test does not assess the effect of upper limb work or activity limitation other than walking [9].

Another test which was proposed to assess essential ADL is Glittre-ADL which may be considered more complete than the 6-minute walk test for assessing individual's functional capacity. It is a performance-based test which involves activities such as sitting and rising from a chair, ascending and descending steps, and movements of the arm while carrying weights. This test was developed to reflect real-life situations better, thus improving the assessment of functional capacity [10]

However, the Glittre-ADL test does not include in-depth and objective assessment of problematic activities involving the upper limbs which are often limited in the elderly [11].

Another option to assess functional status (capacity to perform ADL) is the use of questionnaires. However, the individuals might be influenced by psychological or cognitive factors which may influence the answers [9].

There have been some studies which attempted to assess ADL objectively by using functional tests (like sit-to-stand test). However, functional tests evaluate functional capacity, not ADL performance. Furthermore, functional tests generally do not include a range of specific ADL-like tasks that would represent real life. There has been growing interest on the objective assessment of ADL performance through performance-based protocols.

These protocols encompass the same activities that people perform in their day-to-day life and are thought to provide a more thorough evaluation of ADL performance [12]. Considering the limitations of the available tools used to assess ADL, a new tool known as Londrina ADL protocol was developed by a research group from the State University of Londrina, Brazil [13, 14]. Normative data and the reference ranges for the Londrina ADL protocol for various populations of different ethnic background are currently unavailable.

There is lack of retrievable data on the normative value of the Londrina ADL protocol in healthy subjects in the age group of 40–60 years among the Indian population. The aim of the study is to evaluate the performance of healthy adults using the Londrina ADL test and to obtain normative data and reference equation for the same as per age, gender, and BMI.

## 2. Methodology

### 2.1. Study Population

Healthy subjects with no underlying comorbidities of either gender in the age group 40–60 years were recruited for the study who were referred by a consultant pulmonologist/physician. The entire population was stratified into two groups based on the age groups: 40–49 and 50–60.

### 2.2. Sample Size

The sample size was calculated based on a pilot study of the Londrina ADL protocol time (5 subjects in two groups) with an assumption of 40% subjects having better performance, 15% relative precision, and 95% confidence interval adding 10% nonresponse error. The total sample size comes to 282 using the following formula. *N* = 4pq/d^2^, where *p*, assumption for better performance = 0.4; *q* = 1-pd; nonresponse error = at 15%; *N*, sample size = 282.

### 2.3. Inclusion Criteria

- Healthy adults of either gender, who were referred by a consultant pulmonologist/physician
- Age group: 40 to 60 years
- BMI: 18.0 to 29.5 kg/m

### 2.4. Exclusion Criteria

- History of cardiopulmonary disease
- History of musculoskeletal, neuromuscular, and peripheral vascular diseases which affects the performance of the test
- Use of walking aid
- People who were not able to understand the procedure

### 2.5. Materials

(i) Chair
(ii) Table with a length of 1.2 meters and a width of 0.6 meters
(iii) Weight blocks:
  - 4 objects of 250 grams
  - 4 objects of 500 grams
  - 2 objects of 1 kg
  - 2 objects of 2 kg
(iv) Stand with four adjustable shelves
(v) Clothesline
(vi) 10 dry clothes: 80 to 442 grams
(vii) Weight machine: Equinox BR
(viii) Sphygmomanometer: Diamond Mercurial
(ix) Pulse oxymeter
(x) Stopwatch
(xi) Stadiometer

### 2.6. Study Procedure

A written approval was obtained from the Institutional Ethics Committee and Scientific Committee (IEC). Advertisement was given in local newspaper inviting volunteer participation in the study. Healthy individuals were approached, and the purpose of the study was explained; a written informed consent was obtained from those who were willing to participate. Subjects were screened for inclusion criteria, and eligible subjects were recruited.

Demographic data were collected from each subject, and height and weight were measured using stadiometer and weighing machine. Each subject was then made to perform the Londrina ADL protocol and data were recorded.

#### 2.6.1. Description of Londrina ADL Protocol

**Figure 1:**
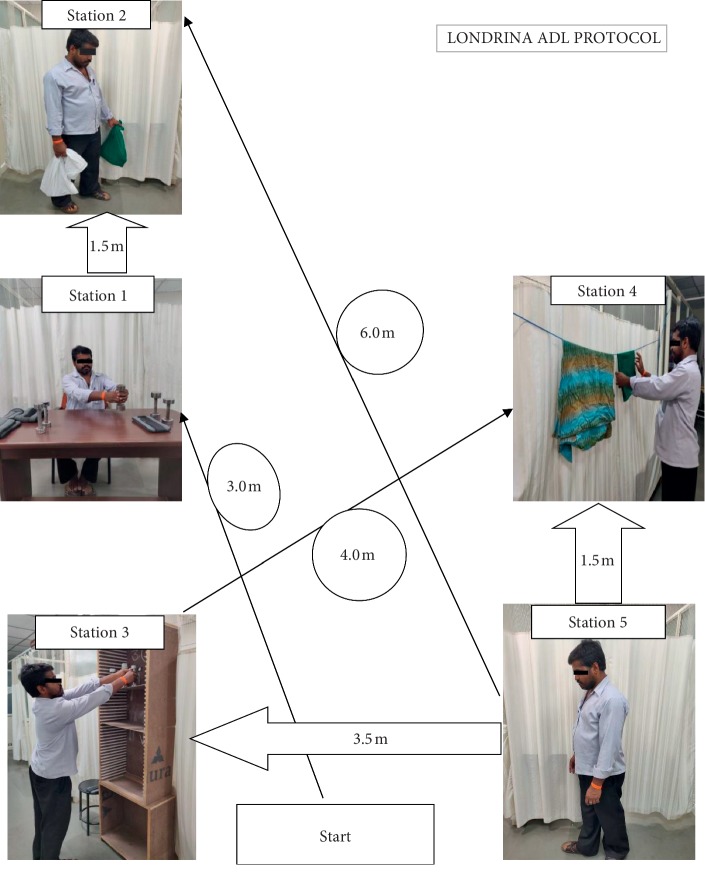
Positioning of activity stations in the Londrina ADL protocol.

The Londrina ADL protocol is composed of 5 activities organized in stations inside a room (minimal dimensions: 6.5 × 5.1 m). The position of the activity stations and the distance between them are shown in Figure 1. The sequence of the stations is as follows:

1. Objects on the table: the participant sits on a chair in front of the table (dimensions: 120 cm (length) ×60 cm (width) with a line separating it into 2 halves (left and right). The table had 10 objects on it (4 objects of 250 g, 4 objects of 500 g, and 2 objects of 1 kg), all together on the left of the table. The subject took the objects, one by one, with both hands and had put them all on the right half of the table. After that, subjects returned the objects in the same way to the left side of the table.
2. Walking with bags: the subject walked over a 6 m line, 3 consecutive times (back and forth, totalling 18 m), carrying 2 bags one in each hand. Inside the bags, there was a load representing 10% of the subject's body weight (5% in each bag).
3. Shelves: the subjects stood in front of 4 shelves, one above the other (distance between the floor and first shelf, 42 cm; distance from one shelf to the next, 45 cm), with a table next to them. On the table, there were 12 objects (4 objects of 250 g, 4 objects of 500 g, 2 objects of 1 kg, and 2 objects of 2 kg). The participant took the objects, one by one, with both hands and placed them on the shelves (without a predetermined order). The subject organized the objects on the shelves in such a way that 3 objects were placed on each shelf. When all of the objects were placed on the shelves, the subject returned them in the same way to the table (without a predetermined order).
4. Clothesline: the subject stood in front of a clothesline positioned at eye level. Basket containing 10 items of clothing (median weight of the items = 122 g (range 80–442 g)) was placed on the ground next to the subject. The subject took all items, one by one, with both hands and hanged them on the clothesline. After hanging all items of clothing, the subject returned them inside the basket, taking them one by one with both hands.
5. Walking: the subject had walked back and forth again on the same 6 m line described in activity 2, 3 consecutive times, but without carrying bags. Participants were instructed to perform the 5 activities at their usual pace.

Before the beginning of the first Londrina ADL protocol, the evaluator demonstrated to the participant the activities of the protocol in the same order in which he/she should perform them. The instructions that were given to the participants are as follows: “Perform these activities as if you were doing them at home in a typical day. You are allowed to stop to rest if you feel it is necessary. We will tell you which station will be the next at the end of each activity.” No encouragement was given during the protocol [13–15].

The time spent to perform the Londrina ADL protocol was registered using a stopwatch, and it was used as the Londrina ADL protocol outcome and the test was performed twice (by the same evaluator) on the same day with a 30 min interval between protocols. The better of 2 performances was taken into consideration. Other measurements before and after the Londrina ADL protocol included: blood pressure, heart rate, and sensation of dyspnea and fatigue (modified Borg scale).

### 2.7. Data Analysis

Statistical analysis was performed using IBM SPSS statistics for Windows version 25.0. Armonk, NY: IBM Corp. Data were expressed as mean ± standard deviation. The association between the time to complete the Londrina ADL protocol and the independent variables was tested by simple linear regression analysis, and stepwise multiple linear regression analysis was used in order to evaluate independent variables explaining the variance in the Londrina ADL protocol. A Bland–Altman plot was also used to visualize agreement between the actual Londrina ADL protocol time and the predicted value.

## 3. Results

A total of 282 healthy individuals were included with 141 healthy adults in the age groups of 40–49 and 50–60 years. Both the groups contained 71 males and 70 females each. The anthropometric data and baseline variables of males and females are presented in Table 1. The time taken for completion of protocol and posttest variables of males and females of trials 1 and 2 are presented in Table 2 and 3.

**Figure 2:**
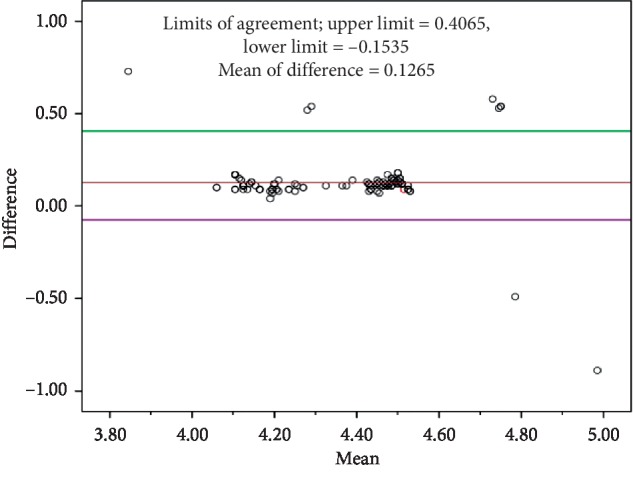
Bland–Altman plot of the difference between the actual and predicted Londrina activities of daily living protocol (LAP) plotted against the mean of actual LAP and predicted LAP. The solid line represents the correlation of the mean with the difference between actual and predicted LAP. The center dotted line shows the mean, and upper and lower dotted lines denote the upper and lower limits, respectively.

The mean time spent by female participants in the age group of 40–49 years is 3.50 ± 0.50 min and in the age group of 50–60 years is 4.25 ± 0.20 min. The mean time spent by male participants in the age group of 40–49 years is 3.73 ± 0.43 min and in the age group of 50–60 years is 4.36 ± 0.18 min. The Londrina ADL protocol was performed twice (1^st^ and 2^nd^ trials) with a 30 min interval. But there is no significant difference found between these two trials with respect to the time taken to complete the protocol (shown in Figure 2) and hemodynamic values.

There was a significant correlation between the performance of the Londrina ADL protocol with age (*r* = 0.74) and height (*r* = 0.54) but not with the weight (*r* = -0.02). In a stepwise multiple linear regression analysis, age and height were selected as Londrina ADL protocol predictors. The Bland–Altman plot (Figure 2) shows the agreement between the actual and predicted Londrina ADL protocol. There was a strong correlation between the average of actual and predicted Londrina ADL protocol duration and the difference between actual and predicted Londrina ADL protocol duration. The ICC value for males and females in the 40 to 50 age group is 0.65 and 0.68, and for males and females in the 50 to 60 age group is 0.75 and 0.87, respectively. The derived reference equation to predict reference values for the Londrina ADL protocol was as follows: equation (1): Londrina ADL predicted = 1.205 + (0.054 × age (years)) + (0.001×height (cm)); equation (2): Londrina ADL predicted = 1.374 + (0.054 × age (years)) + (−0.003 × BMI). Only these two equations were found to be statically significant.

## 4. Discussion

The present study aimed at providing normative values of Londrina ADL protocol in healthy individuals among the Indian population. A total of 71 males and 70 females were recruited in both the age groups (40–49 and 50–60 years). In the age group of 40–49 years, the average time taken for completion of test by females is 3.50 ± 0.50 min and males have performed the test in 3.73 ± 0.43 min. Similarly, in the age group of 50–60 years, males have performed the test in 4.36 ± 0.18 min and females in 4.25 ± 0.20 min.

We hypothesized that variables such as age, gender, height, weight, and BMI would be the predictors of the time taken to complete the Londrina ADL protocol as these factors can influence the individual's performance on functional tests. There is no significant difference found between males and females in both the age groups in view of the time taken to complete the protocol. Despite the protocol including activities that are more common to women's routine due to the presence of domestic tasks, males can also perform similar movements during work activities [13]. The Londrina ADL protocol was performed twice (1^st^ and 2^nd^ trial), and the obtained results show that the majority of subjects performed their best in 2^nd^ trial which can be attributed to the learning effect.

In a similar study on older adults above 50 years of age carried out on a Brazilian population by Paes et al., it was found that the average time for the completion of the test was 5.06 ± 0.51 minutes. In our study, we found that the completion of the test by the same age group in the Indian population took 1 minute less. We found 1 minute lesser for the completion of the test in the same age group. This variability may be influenced by inclusion of other factors, such as age, health conditions, low level of physical activity, education, social class, income, life history, personality features, speed of habitual walking, or cultural aspects related to lifestyle, mood, attitude, and motivation of the subject and/or technician, and hence, older individuals are reportedly less physically active which represents the potential risk for decreased mobility. It is known that frailty is associated with functionality and other factors such as fatigue, multiple health problems, vision, hearing, cognition, and psychological disturbances (depression and anxiety) [13, 16].

The Londrina ADL protocol provides the possibility of having a standardized method to assess different outcomes during the performance of ADL. This is a valid test because a correlation was found between movement intensity in daily life and during the protocol, which indicates a correlation between the individual's real life and the individual's performance during ADL [14, 17]. The Londrina ADL protocol correlated moderately with upper and lower limb function, mobility, balance, and level of physical activity in daily life [13].

A study done by Sant Anna et al. showed that subjects with COPD took an average time of 6.31 ± 0.18 to complete the test, which is 2 minutes greater when compared with healthy individuals. The disparity was due to the presence of pulmonary dysfunction which is progressive obstruction of the airflow. Extrapulmonary features, such as abnormalities of skeletal muscles (respiratory and limb), caused by aberrations in gas exchange, presence of malnutrition, use of drugs, state of physical inactivity, respiratory tract malformation, will negatively influence the subject's exercise capacity and performance of daily life activities [14, 18].

In our study, during the Londrina ADL protocol, there was a mild increase in heart rate, respiratory rate, dyspnea, and fatigue in healthy subjects when compared before and after the test, which may be because of the multiple activities that are included in the Londrina ADL protocol as the tasks require different energy expenditure. It may be because during the exercise there is an increase in metabolic demand because of which there is an increase in cardiac output to supply the peripheral oxygen need. The increase in the initial HR is due to the removal of parasympathetic tone, and at the higher rates of work, this increase is due to the sympathetic nervous system [19].

A study was done on the Brazilian population by Paes et al, to evaluate the episodes of oxygen desaturation during Londrina ADL protocol in patients with COPD. And they found that there is a moderate correlation of (*r* = 0.45) episodes of desaturation (≥4%) in daily life especially with SPO2 being under 88%. As the Londrina ADL protocol represents better performance in day-to-day activities, the hypothesis which was given to explain the decrease in oxygen saturation in daily life was the influence of blood perfusion of body extremities. The initiation of respiratory symptoms could be better reviewed when patients report how they feel during the performance of routine activities in daily life [15].

The Londrina ADL protocol represents better performance in day-to-day activities, the hypothesis which was given to explain the decrease in oxygen saturation in daily life could be influence of the blood perfusion of body extremities and the initiation of the respiratory symptoms could be better reflected when patients reported how they feel in daily life during their day-to-day activities [15].

The main limitations of the study were as follows: we have studied the normative values in the age groups of 40–60, as most of the pulmonary conditions are seen in these age groups. We did not study the normative values in the age groups of 30–39 and 60–70 years. We also did not study the influence of other factors such as balance, peripheral muscle strength, and cognition and behavior which have an influence on the time taken to complete the test. We did not analyze the relationship between Londrina ADL protocol performance and other limiting factors like fatigability or breathlessness within each age group.

Future studies can be done on pulmonary conditions and cardiovascular diseases to assess the performance of ADL and compare with our findings as reference values to determine the reliability of the equations.

## 5. Conclusion

In our study, we concluded that the reference value for Londrina ADL protocol in healthy individuals in the age group of 40–49 years in females is 3.50 ± 0.50 min and in males is 3.73 ± 0.43 min, and in the age group of 50–60 in females is 4.25 ± 0.20 min and in males is 4.36 ± 0.18 min.

## Figures and Tables

**Table 1:** The anthropometric data and baseline values of hemodynamic parameters, rate of perceived exertion, and fatigue of the Londrina ADL protocol in males and females (mean ± SD).

Variables	40–49 (*n* = 141), mean ± SD		50–60 (*n* = 141), mean ± SD	
	Males	Females	Males	Females
Age (years)	44.30 ± 2.81	44.4 ± 2.79	55.11 ± 2.95	55.03 ± 3.04
Height (cm)	161.18 ± 3.50	157.69 ± 3.97	162.61 ± 3.15	157.99 ± 2.94
Weight (kg)	69.14 ± 2.66	66.86 ± 3.97	70.23 ± 2.61	66.63 ± 3.32
BMI (kg/m^2^)	26.42 ± 0.81	26.83 ± 1.28	26.55 ± 1.01	26.59 ± 1.12
Heart rate (beats per min)	77.49 ± 4.05	77.90 ± 4.27	79.41 ± 2.99	79.24 ± 3.04
Respiratory rate (breathes per min)	20.66 ± 1.85	20.33 ± 1.71	20.04 ± 1.02	20.25 ± 1.06
O2 saturation (%)	98.21 ± 0.82	98.47 ± 0.69	97.81 ± 0.95	97.72 ± 0.95
Systolic blood pressure (mmHg)	123.24 ± 6.27	123.29 ± 7.16	124.14 ± 7.12	124.08 ± 6.88
Diastolic blood pressure (mmHg)	83.80 ± 6.40	86.29 ± 7.83	88.29 ± 7.79	87.04 ± 7.25
Dyspnea (Borg scale)	0.5 ± 0.10	0.5 ± 0.72	0.48 ± 0.16	0.5 ± 0.08
Fatigue (Borg scale)	0.44 ± 0.11	0.5 ± 0.69	0.5 ± 0.15	0.48 ± 0.05

**Table 2:** The time taken for completion of the Londrina ADL protocol and posttest values of hemodynamic parameters, rate of perceived exertion, and fatigue in males (mean ± SD).

Variables		40–49 (*n* = 71)		*p* value	50–60 (*n* = 71)		*p* value
		1^st^ trial	2^nd^ trial		1^st^ trial	2^nd^ trial	
Londrina ADL time (min)	Mean ± SD	4.00 ± 0.21	3.73 ± 0.43	0.00	4.49 ± 0.18	4.36 ± 0.18	0.00
	Minimum and maximum values (95% CI)	3.53 – 4.30	3.00 – 4.00		4.18 – 5.02	4.02 – 5.43	
Heart rate (beats per min)		81.0 ± 3.95	81.07 ± 3.94	0.00	82.60 ± 3.48	82.62 ± 3.49	0.00
Respiratory rate (breathes per min)		23.44 ± 1.69	23.45 ± 1.67	0.00	22.73 ± 1.16	22.44 ± 1.18	0.00
O2 saturation (%)		97.87 ± 0.71	97.86 ± 0.16	0.00	97.69 ± 0.91	97.67 ± 0.92	0.00
Systolic blood pressure (mmHg)		123.38 ± 6.31	123.37 ± 6.34	0.08	124.86 ± 6.75	124.84 ± 6.72	0.08
Diastolic blood pressure (mmHg)		88.73 ± 6.07	688.73 ± 6.05	0.00	91.29 ± 8.15	91.28 ± 8.14	0.00
Dyspnea (Borg scale)		0.5 ± 0.13	0.5 ± 0.15	0.00	0.48 ± 0.20	0.48 ± 0.21	0.00
Fatigue (Borg scale)		0.46 ± 0.16	0.50 ± 0.19	0.00	0.5 ± 0.19	0.5 ± 0.20	0.00

**Table 3:** The time taken for completion of the Londrina ADL protocol and posttest values of hemodynamic parameters, rate of perceived exertion, and fatigue in females (mean ± SD).

Variables		40–49 (*n* = 70)		*p* value	50–60 (*n* = 70)		*p* value
		1^st^ trial	2^nd^ trial		1^st^ trial	2^nd^ trial	
Londrina ADL time (min)	Mean±SD	3.87 ± 0.27	3.50 ± 0.50	0.00	4.37 ± 0.16	4.25 ± 0.20	0.00
	Minimum and maximum values (95% CI)	3.40 – 4.16	3.00 – 4.00		4.11 – 4.58	3.48 – 5.03	
Heart rate (beats per min)		81.10 ± 4.27	81.12 ± 4.28	0.00	4.37 ± 0.16	4.25 ± 0.20	0.00
Respiratory rate (breathes per min)		22.80 ± 1.34	22.82 ± 1.32	0.00	22.90 ± 1.12	22.92 ± 1.11	0.00
O2 saturation (%)		98.09 ± 0.55	98.07 ± 0.53	0.00	97.59 ± 0.88	97.59 ± 0.87	0.00
Systolic blood pressure (mmHg)		123.71 ± 7.05	123.72 ± 7.07	0.08	124.51 ± 6.71	124.52 ± 6.71	0.08
Diastolic blood pressure (mmHg)		90.29 ± 7.60	90.27 ± 7.58	0.00	90.85 ± 7.31	90.81 ± 7.29	0.00
Dyspnea (Borg scale)		0.5 ± 0.70	0.5 ± 0.74	0.00	0.5 ± 0.08	0.5 ± 0.10	0.00
Fatigue (Borg scale)		0.5 ± 0.63	0.5 ± 0.69	0.00	0.48 ± 0.05	0.48 ± 0.08	0.00

## Data Availability

The data used to support the findings of this study are available from the corresponding author upon request.
